# MAPKDB: A MAP kinase database for signal transduction element identification

**DOI:** 10.6026/97320630015338

**Published:** 2018-05-15

**Authors:** Hamdan Ali Alshehri, Nadim W Alkharouf, Omar Darwish, Brant T. McNeece, Vincent P. Klink

**Affiliations:** 1 Department of Computer and Information Sciences, Towson University, Towson, MD 21252, USA; 2 Department of Mathematics and Computer Science, Texas Woman’s University, Denton, TX 76204; 3 Department of Biological Sciences, Mississippi State University, Mississippi State, MS 39762, USA; 4 Center for Computational Sciences High Performance Computing Collaboratory, Mississippi State University, Mississippi State, MS 39762, USA

**Keywords:** MAPKDB, MAP kinase, database

## Abstract

The mitogen activated protein kinase (MAPK) cascade is a central signal transduction platform, ubiquitous within the eukaryotes. MAPKs function prominently in different essential cellular processes such as proliferation, differentiation, survival and defense to pathogen attack. The 32 MAPKs of Glycine max (soybean) have been examined functionally to determine if they have any defense role, focusing in on infection by the plant-parasitic nematode Heterodera glycines. Of these 32 MAPKs, 9 have been shown to have a defense function. Hence, the Mitogen Activated Protein Kinase database (MAPKDB) has been developed to assist in such research. The MAPKDB allows users to search the annotations with sequence data for G. max transgenic lines undergoing overexpression (OE) or RNA interference (RNAi) of its defense map kinases. These defense MAPKs include map kinase 2 (MPK2), MPK3, MPK4, MPK5, MPK6, MPK13, MPK16, and MPK20. The database also contains data analysis information for each sample that helps to detect the differential expression of the genes identified within these samples. The database also contains data for each sample that helps to detect the differential expression of the genes identified within these samples. The database has been developed to manage G. max MAPK sequences with sequence alignment for 18 different samples along with two additional OE and RNAi control experiments for a total of 20.

## Background

Living organisms are constantly inundated with stimuli of biotic or abiotic nature. These signals are transduced through transduction cascades, allowing them to survive. The mitogen activated protein kinase (MAPK) cascade is a central signal transduction platform that is ubiquitous in the eukaryotes. The cascade is three tiered, transducing input information through a stepwise series of phosphorylation events, leading to an appropriate output response having high fidelity 1. Consequently, it has been stated that the MAPK platform functions as a cooperative enzyme, switching cells from one distinct state to another 2. In less usual circumstances, MAPKs have been observed to function independently of both MEKKs (MAPK/ERK Kinase Kinase) and MEKs (MAPK/ERK Kinase) by auto phosphorylation of these proteins 3. Therefore, there are many things that remain to become understood regarding the function of MAPK signalling, particularly in plants. Some of the earlier studies on MAPK signalling that have been done in plants have benefitted from the diploid genetic model Arabidopsis thaliana, owing to its sequenced genome 4. The analyses have identified 80 MAPKKKs that would be expected to transduce signal information through its 10 MAPKKs and then 20 MAPKs (MPKs), ultimately leading to various output responses 4.While a number of analyses have been done in A. Thaliana with regard to their MPKs, these studies have largely focused in on those functioning in defense to pathogens with the majority focusing in on MPK3 5. The absence of a comprehensive functional analysis of a MAPK gene family in any biological system led to the characterization the MAPKs in the model crop system Glycine max (soybean) 6.

The 32 MAPKs of G. max have been examined functionally to determine if they have any role in defending to the plant parasitic nematode Heterodera glycines 7. The combination of gene overexpression (OE) and RNAi experiments for all 32 G. max MAPKs have revealed that nine of them have a defense function, impairing H. glycines parasitism 8. The G. Max defense MAPKs includes homologs of A. thaliana MPK2, MPK3, MPK4, MPK5, MPK6, MPK13, MPK16 and MPK20. These nine MAPKs became the basis of the RNA-seq analysis presented herein. In the analysis of McNeece (2019), each of its defense MAPKs had been characterized regarding their expression in relation to each other and a series of already proven defense genes that function in the G. max-H. Glycines pathosytem^6^. In those experiments, pathogen associated molecular pattern (PAMP) triggered immunity (PTI) has been examined using quantitative PCR (qPCR) probes targeting ENHANCED DISEASE SUSCEPTIBILITY1 (EDS1) and LESION SIMULATING DISEASE1 (LSD1)^9^. Effector-triggered immunity (ETI) has been studied by examining harpin in relation to MAPK gene expression. PTI and ETI had been examined further focusing in on G. max homologs of the PTI gene NON-RACE SPECIFIC DISEASE RESISTANCE 1/HARPIN INDUCED1 (NDR1/HIN1) while ETI had been focused in on in analyses of BOTRYTIS INDUCED KINASE1 (BIK1) 9. The proven downstream defense genes that had been included in the analysis had been those composing the PTI and ETI signal transduction cascades, alpha hydroxyl nitrile biogenesis, and cyanide metabolism, the 20S membrane fusion particle, carbon and hemi-cellulose metabolism, an ABC-G type transporter and PATHOGENESIS RELATED1 (PR1) 10.

## Methodology

### Construction of website database:

The Mitogen Activated Protein Kinase database (MAPKDB) has been designed and implemented to manage annotations and sequencing of G. max MAPK, allowing users to implement designed queries. The MAPKDB database stores essential data relating to G. max MAPK-OE and RNAi experiments and retrieves the data based on gene identification (geneID). The database stores descriptions of each gene obtained from the study samples, eukaryotic orthologous groups (KOG), gene ontology (GO) assignments, and protein families (PFAM). MAPKDB has been designed, implemented, and hosted using Microsoft SQL Server 2016 Enterprise Edition. The MAPKDB web application has been designed and implemented using ASP.NET with C# programming language which relies on the integrated development environment Microsoft Visual Studio 2017. In addition, the operating system that has been used for the server is Microsoft Windows Server 2012 and Internet Information Services version 7.0. The bioinformatics server at Towson University in Towson, MD, USA hosts the database and website of MAPKDB. We have developed a user-friendly database-driven website that allows users to access all the stored data. Users can browse, search and download the data using gene IDs or descriptions. In addition, users can compare the differential gene expression results in the different samples.

## Utility and Discussion:

RNA sequencing data allows one to obtain a very precise analysis of the expression of genes in a given transcriptome. In the experiment presented here, we have obtained between 650 and 700 million sequence reads from the nine different G. max MAPK-OE samples and nine different G. max MAPK RNAi samples, resulting in data size of about 850GB. We used the Bowtie2 11 sequence alignment software to align the sequences obtained from the G. max MAPK-OE and RNAi samples to the soybean reference genome sequence (which version of the genome?) (https://phytozome.jgi.doe.gov/pz/portal.html). A summary of the RNA sequences alignment results that was produced from Bowtie2 is shown in Table 1. After alignment we applied feature Counts 12 to count the number of mapped reads to each soybean gene transcript. DEGSeq (https://bioconductor.org/ packages/release /bioc/html/ DEGseq.html) was then used for differential gene expression analysis of the samples. Experimental samples were compared to control samples in both the MAPK-OE and RNAi groups.

**Figure 1:**
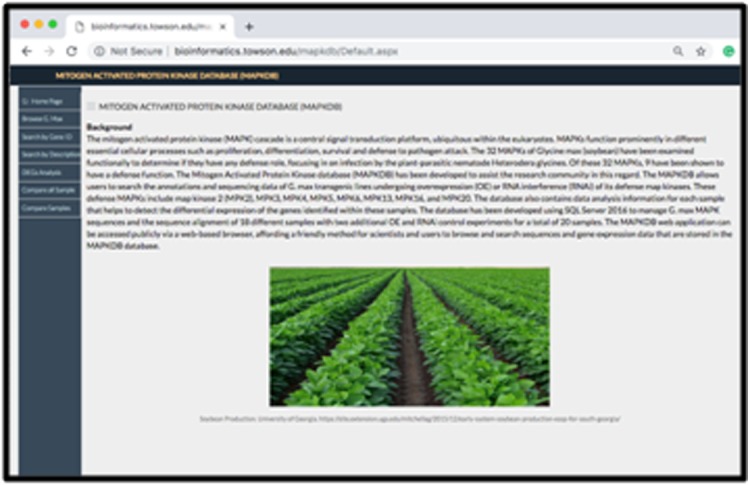
A snapshot of the MAPKDB main web page

The user interface (Figure 1) of MAPKDB provides users with the following functionalities:

### Browse:

The user can browse any sample that has been stored in MAPKDB database. The Browse.aspx web page shows a table that can be exported to an Excel file that contains many sequence attributes such as geneID, transcript identification, description, KOG, gene ontology and PFAM. Also, users can see the details of each transcript that shows the transcript ID, description, and FASTA sequence when the plus image is clicked (at the beginning of the row).

### Search:

The MAPKDB allows users to search the sequence information in two separate ways. Users can search sequence data through gene ID, or its accompanying description. When users search by gene ID, the exact gene ID has to be entered in the text box to get the matched result. Alternatively, partial characters or text can be entered into searches for genes if the user searches by description. All of the different searches return their query results in a nice table that also shows the sequence data.

### Gene expression results:

**Figure 2:**
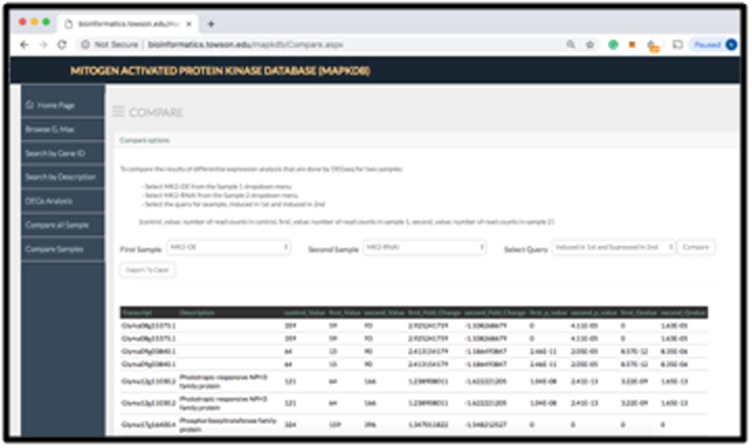
A snapshot of the comparing samples page

Users can select a specific sample and retrieve a list of all the transcripts in that sample with their accompanying differential gene expression results (output from DEGSeq). The user can also narrow down the results by searching for specific gene(s) in the analysis. This is done by typing the exact gene ID(s) in the text box to get the gene information that matches those genes. In addition, the user can narrow down the results by searching for gene descriptions or parts of a description. This task is accomplished by typing the partial character(s) in the text box that exists beside the Search by Description identifier (Figure 2).

### Comparing all samples:

The MAPKDB empowers users to compare any two samples. The search will compare the differential expression analysis results from each of the selected samples (MAPK-OE or RNAi experiments). In this web page, the web application enables users to retrieve the genes that are induced or suppressed in the first selected sample and induced or suppressed in the second sample. This search allows allow users to compare samples with their controls. All of these queries return their results in a user-friendly table and the user has the ability to download the data to an excel file.

## Conflict of Interest

Authors declare no conflict of interest.

## Figures and Tables

**Table 1:** Shows the result of Bowtie2 alignment to soybean gene transcripts

Sample	Overall Alignment rate (%)
MK13-1-OE-R1	85.16
MK13-1-RNAI-R1	83.85
MK16-4-OE-R1	82.23
MK16-4-RNAI-R1	82.1
MK2-OE-R1	83.48
MK2-RNAI-R1	82.33
MK20-2-OE-R1	85.59
MK20-2-RNAI-R1	87.33
MK3-1-OE-R1	84.08
MK3-1-RNAI--R1	83.83
MK3-2-OE-R1	85.39
MK3-2-RNAI-R1	82.58
MK4-1-OE-R1	84.06
MK4-1-RNAI-R1	82.46
MK5-3-OE-R1	85.65
